# Learning as a shield against death: a 10-day daily diary study examining mortality salience and academic engagement among college students

**DOI:** 10.3389/fpsyg.2026.1727723

**Published:** 2026-01-27

**Authors:** Yaqin Yan, Qing Xie, Ji Lai

**Affiliations:** 1 Department of Student Affairs, Hunan First Normal University, Changsha, China; 2 Department of Management, Hunan Police Academy, Changsha, China

**Keywords:** academic engagement, compensatory control model, daily diary study, death anxiety, mortality salience, personal control, terror management theory

## Abstract

Academic engagement is a critical determinant of success and wellbeing for college students. While previous research has identified the influence of stable environmental and individual factors, it has largely overlooked the impact of dynamic, daily psychological experiences. Moreover, mortality salience can be triggered by common experiences such as pandemics or accidents, yet its link to academic engagement remains unexplored. Grounded in Terror Management Theory (TMT) and Compensatory Control Model (CCM), this study employed a 10-day daily diary study with 102 Chinese undergraduates to investigate how daily mortality salience influences academic engagement. Results from a multilevel path analysis revealed significant, though modest, positive indirect associations between daily mortality salience and academic engagement both through death anxiety and personal control. These findings demonstrate that academic engagement can serve as a compensatory mechanism to manage existential threat. This study not only bridges a theoretical gap by integrating TMT and CCM but also provides practical insights, suggesting that educators can enhance engagement by fostering a sense of meaning and personal control in the learning environment.

## 1 Introduction

Academic engagement, which refers to students’ investment of effort, attention, and persistence in learning activities, is a critical predictor of academic performance and personal development (Zhang et al., 2021; King and Gaerlan, 2014). Consequently, it has garnered significant scholarly attention. However, prior research has predominantly adopted a static perspective, focusing on the influence of stable factors such as educational context, family environment, and personal traits, while largely overlooking the dynamic role of daily stimuli and experiences (Mou et al., 2022; Alt, 2015; Liu et al., 2014; Upadyaya and Salmela-Aro, 2013). Meanwhile, in the post-pandemic era, an increasing number of researchers are focusing on the effects of mortality salience—the conscious awareness of one’s own death—on individuals (Seoin et al., 2021; Evers et al., 2021). Beyond COVID-19, daily life frequently exposes individuals to various death-related cues, such as accidents, news reports, films, or funerals. This raises an important question: How do these daily encounters with mortality influence the academic engagement of college students?

Terror Management Theory (TMT; Greenberg et al., 1986) and Compensatory Control Model (CCM; Kay et al., 2008) provide two dynamic frameworks for analyzing this issue, as they elucidate how immediate psychological responses to threats can motivate behavior. Research based on these theories indicates that mortality salience is a potent motivator of human behavior, prompting individuals to bolster their sense of self-worth and personal control (Greenberg et al., 1992; Fritsche et al., 2008). From this theoretical standpoint, mortality salience could potentially enhance undergraduates’ academic engagement. Nevertheless, this proposed relationship and its underlying psychological mechanisms—specifically, the proposed parallel mediating roles of death anxiety and personal control—have not been empirically examined.

Employing a daily diary study, the current research aims to investigate the impact of daily mortality salience on college students’ academic engagement and its underlying mechanisms. The insights from this study are expected to inform educational practices aimed at promoting academic engagement, while also enriching the psychological literature on how individuals cope with mortality awareness.

### 1.1 Terror management theory: the role of death anxiety

Grounded in the existential insights of Becker (1973), TMT posits that humans are uniquely aware of their own mortality (Greenberg et al., 1986). This awareness creates a fundamental conflict between the inevitability of death and the biological instinct for survival. When mortality is made salient, it triggers death anxiety—a state of distress and apprehension related to death-related thoughts (Pyszczynski et al., 2004; Tomer, 1992). Substantial evidence confirms that mortality salience elevates death anxiety (Juhl and Routledge, 2016; Belmi and Pfeffer, 2016; Zaleskiewicz et al., 2013). To alleviate this existential threat, individuals engage in behaviors that enhance self-worth and meaning, thereby achieving a sense of symbolic immortality. Symbolic immortality involves becoming part of something larger and more enduring than the self (Vail et al., 2020; Lifshin et al., 2021). As Lifton proposed, both the intellectual mode (creating lasting works or legacies) and the experiential mode (immersing oneself in meaningful activities) can facilitate this symbolic transcendence (Lifton, 1973).

Academic engagement embodies both modes: it is an experiential immersion in learning that can produce intellectual legacies through academic achievement (Topçu et al., 2018). Prior research supports this view, demonstrating that mortality reminders promote self-transcendence, creativity, and work engagement (Joireman, 2005; Routledge et al., 2010; Hu et al., 2020). In a particularly relevant finding, Zestcott et al. (2016) showed that mortality salience enhanced basketball performance among undergraduates, suggesting that achievement-oriented activities can serve as anxiety-buffering mechanisms.

Consequently, we hypothesize that daily mortality salience fuels academic engagement by heightening death anxiety, thereby demonstrating a within-person mediation process.

*Hypothesis 1*: Daily mortality salience is positively associated with academic engagement through the mediating role of death anxiety at the within-person level.

### 1.2 Compensatory control model: the role of personal control

While TMT primarily addresses the impact of death’s inevitability, the profound unpredictability of death—when, how, and where it occurs—also constitutes a significant psychological threat (Dechesne and Kruglanski, 2004). This uncertainty is captured by the adage that we never know whether tomorrow or a sudden accident will arrive first. Empirical evidence confirms that reminders of death can undermine an individual’s sense of personal control (Fritsche et al., 2008; Hohman and Hogg, 2015).

CCM provides a framework for understanding responses to this threat. CCM posits that individuals are motivated to maintain a belief in an orderly and manageable world. When this sense of control is threatened, people engage in restorative actions to reclaim it (Kay et al., 2008). This is evidenced by findings that mortality salience intensifies the desire for fairness, order, and certainty (Kay et al., 2009, 2010; Van den Bos, 2001).

We argue that academic engagement serves as one such compensatory strategy. For students, sustained focus and adherence to learning represent a direct assertion of personal control (Rock, 2005). Furthermore, the knowledge and skills gained through academic engagement enhance one’s capacity to navigate future uncertainties (Merino-Tejedor et al., 2016). Success in academics also brings favorable evaluations and strengthens social bonds, thereby further reinforcing personal control. Supporting this logic, Yang et al. (2019) demonstrated that diminished personal control directly leads to improved performance on self-esteem-relevant tasks.

Consequently, we hypothesize that on days when students experience higher mortality salience, their sense of personal control is threatened, which in turn motivates greater academic engagement as a compensatory strategy.

*Hypothesis 2*: Daily mortality salience is positively associated with academic engagement through the mediating role of personal control at the within-person level.

### 1.3 Current study

Both TMT and the CCM emphasize dynamic, state-level psychological processes. TMT posits that mortality salience triggers an immediate state of death anxiety, which motivates defenses. Similarly, CCM suggests that threats to personal control elicit immediate compensatory behaviors to restore order. These are not static traits but fluctuate with daily experiences. A diary methodology is uniquely suited to capture these within-person, day-to-day fluctuations in mortality salience, its concomitant psychological states (anxiety, control), and their behavioral consequences (academic engagement), thereby testing the core, dynamic propositions of these theories with high ecological validity.

**Figure 1:**
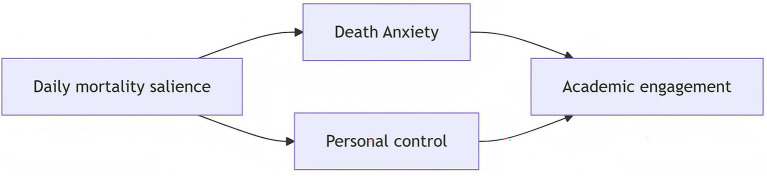
The research framework.

Grounded in the dual nature of death—its inevitability and unpredictability—this research integrates TMT with CCM to examine the impact of mortality salience on college students’ academic engagement, specifically through the parallel mediators of death anxiety and personal control. While previous research has predominantly relied on laboratory experiments using mortality imagination tasks, real-world cues (e.g., news, cemetery) have also been shown to trigger mortality salience effects (Das et al., 2009; Jonas et al., 2002). To enhance ecological validity, the present study adopts a daily experience sampling design to investigate how naturally occurring mortality salience influences academic engagement. The comprehensive research framework is illustrated in Figure 1.

## 2 Materials and methods

### 2.1 Participants and procedure

A daily diary study was administered in October 2024 to a convenience sample of 104 Chinese undergraduates. Over 10 weekdays across two consecutive weeks, participants completed nightly surveys at 8:00 p.m. measuring mortality salience, death anxiety, personal control, and academic engagement, with demographics assessed on the final day. Following the attrition of two participants, the final sample of 102 students provided 1,006 daily responses (98.6% compliance). The sample had a mean age of 21.50 years (SD = 2.07) and was 70.6% female. Participants received a modest compensation of 15 RMB (approximately 2 USD) upon completion of all 10 daily surveys. This token of appreciation was intended to acknowledge their time commitment and to support recruitment and retention, particularly for a multi-day study. The compensation was not contingent on the content of their responses. Post-hoc checks were conducted for response patterns indicative of insufficient effort (e.g., patterned responding, aberrant response times, or internal inconsistencies). No widespread issues of insufficient effort responding were detected.

### 2.2 Measures and analysis

#### 2.2.1 Measures

Table 1 presents the scales used in our research. Mortality salience was assessed with two items adapted from Hu et al. (2020), who successfully used this brief measure in a diary study during the COVID-19 pandemic. Participants rated these items on a 7-point Likert scale ranging from 1 (not at all) to 7 (very much). This operationalization captures the core, face-valid manifestation of the construct in daily life: (1) internal, cognitive-affective awareness (“Today, I had thoughts or feelings about death in my mind”), and (2) external, cue-driven exposure (“Today, there were news, events or experiences that reminded me of death”). This two-item approach balances comprehensiveness with the brevity required for daily repeated measurements, minimizing participant burden and fatigue across 10 days. Crucially, by asking participants to report on their experiences ‘Today’, these items are designed to be sensitive to the day-to-day variations in mortality-related awareness that are central to our research questions. Death anxiety was measured using four items from Belmi and Pfeffer (2016) (e.g., “Today, I worried about death”); personal control was evaluated with three items adapted from Fritsche et al. (2008) (e.g., “Today, I felt helpless”; reverse scored); and academic engagement was gauged using four items adapted from Schaufeli et al. (2002) (e.g., “Today, I felt energetic when studying”). These three constructs were rated on a 7-point Likert scale ranging from 1 (strongly disagree) to 7 (strongly agree). All scales demonstrated good internal consistency in the present study (*α* = 0.91 for mortality salience; *α* = 0.94 for death anxiety; *α* = 0.90 for personal control; *α* = 0.95 for academic engagement).

**Table 1:** Measures in current study.

Variable	Number of items	Measure	Items	Scale anchor
Mortality salience	2	Adapted from Hu et al. (2020)	Today, I had thoughts or feelings about death in my mind; Today, there were news, events or experiences that remind me of death.	Not at all – Very much (7-point)
Death anxiety	4	Belmi and Pfeffer (2016)	Today, I was very much afraid to die; Today, I felt worried about my death; Today, death scared me; Today, I worried about death.	Strongly disagree – Strongly agree (7-point)
Personal control	3	Adapted from Fritsche et al. (2008)	Today, I felt helpless; Today, I felt powerless; Today, I felt lack of control.	Strongly disagree – Strongly agree (7-point)
Academic engagement	4	Adapted from Schaufeli et al. (2002)	Today, I was enthusiastic about studying; Today, I felt energetic when studying; Today, I felt very happy when I devoted myself to study; Today, I felt that time passed quickly when studying.	Strongly disagree – Strongly agree (7-point)

It should be noted that, the use of reverse-scored items assessing helplessness (e.g., ‘I felt helpless’) to operationalize personal control is grounded in established theory. The core affective-cognitive experience of helplessness is the direct antithesis of perceived control, a central tenet of learned helplessness theory (Seligman, 1972). When individuals feel helpless, powerless, or lacking control, they are explicitly reporting a state of low perceived agency. While these items have an affective component, they are distinct from measures of general emotional distress (e.g., sadness, anxiety) as they specifically target appraisals of one’s capacity to influence events (e.g., ‘I felt lack of control’). This operationalization is widely used and validated in existential and motivational psychology research to capture state-level fluctuations in personal control (e.g., Fritsche et al., 2008; Li et al., 2021).

We controlled for students’ age and gender (0 = male, 1 = female) at the between-person level, as prior research suggests these demographics may correlate with mortality salience and activity engagement (e.g., Belmi and Pfeffer, 2016; Roberts and Maxfield, 2019). Additionally, to account for temporal carry-over and more rigorously isolate the day-to-day effect of our predictors, we followed the analytic approach common in daily diary studies (Hu et al., 2020) and included the previous day’s level of academic engagement as a within-person (Level-1) predictor in our multilevel path models.

#### 2.2.2 Data analysis

The data analysis proceeded in three stages. First, we computed the descriptive statistics and correlations among the variables. Second, we performed a confirmatory factor analysis (CFA) to examine the discriminant validity of the measures. Third, we tested our hypothesized model using multilevel path analysis with Bayesian estimation (Zyphur and Oswald, 2015). We employed Bayesian estimation for several reasons aligned with the characteristics of our data and model: (1) It is particularly well-suited for multilevel models with a moderately small number of Level-2 units (*N* = 102 individuals), providing stable estimates for complex random-effects structures; (2) It directly yields the full posterior distribution of all parameters, allowing us to obtain credible intervals (CrIs) for point estimates—including the indirect effects—without relying on normality assumptions, which are often violated for product terms in mediation analysis; (3) It robustly supports the estimation of our within-person parallel mediation model and accommodates potential non-normality in the daily-level data. We specified weakly informative (default) priors for all model parameters, allowing the data to dominate the posterior distributions. Model convergence was assessed using the Potential Scale Reduction (PSR, or R-hat) statistic, with all parameter PSR values < 1.01, indicating good convergence. Within-person predictors were person-mean centered, and between-person predictors were grand-mean centered. All analyses were conducted in Mplus 8 (Muthén and Muthén, 2017).

## 3 Results

Table 2 presents results of descriptive statistics and correlations among the variables. We also report the intraclass correlation coefficients (*ICC*1) and scale reliabilities. Then we conducted a multilevel confirmatory factor analysis with our focus variables. This four-factor model demonstrated an adequate fit to the data: *χ*^2^(118) = 389.00, CFI = 0.94, TLI = 0.90, RMSEA = 0.05, SRMR_within_ = 0.04, SRMR_between_ = 0.03.

**Table 2:** Means, standard deviations, and correlations among variables in current study.

Variables	*M*	SD	ICC1	1	2	3	4	5	6	7
Within-person (Level 1)										
Mortality salience	2.30	0.11	0.36	**(0.91)**	0.65^***^	−0.56^***^	0.09	0.10	0.30**	−0.18
Death anxiety	2.05	0.11	0.59	**0.47** ^***^	**(0.94)**	−0.54^***^	0.03	0.03	0.03	0.10
Personal control	3.84	0.13	0.56	**−0.31** ^***^	−0.31^***^	**(0.90)**	−0.25^*^	−0.26^*^	−0.25^*^	0.10
Academic engagement	4.41	0.14	0.71	**0.14** ^**^	**0.17** ^**^	**−0.15** ^**^	**(0.95)**	0.99^***^	0.18	−0.06
Academic engagement (*p*)	4.40	0.14	0.71	−0.02	0.00	−0.02	0.03		0.19	−0.07
Between-person (Level 2)										
Gender	0.70	0.05	–	–	–	–	–	–		−0.21^*^
Age	21.50	0.20	–	–	–	–	–	–	–	

*N* = 102, *n* = 1,006. Within-person correlations are below the diagonal, and between-person correlations are above the diagonal. Scale reliabilities (Cronbach’s alpha coefficients) are reported along the diagonal in parentheses. ICC1 = intraclass correlation coefficient. ICC1 is calculated as: (between-person variance)/(between-person variance + within-person variance). Gender (male = 0, female = 1). Academic engagement (*p*) = academic engagement on the previous day. The bold formatting in Table 2 highlights the scale reliabilities and the within-person correlation coefficients tested in the model. ^*^p < 0.05, ^**^p < 0.01, ^***^p < 0.001 (two-tailed).

As shown in Tables 3 and 4, daily mortality salience was positively related to death anxiety (*B* = 0.33, SE = 0.02, 95% CI [0.286, 0.366]). Death anxiety was positively related to academic engagement (*B* = 0.12, SE = 0.04, 95% CI [0.046, 0.193]). The mediating role of death anxiety on the relationship between mortality salience and academic engagement was significant (Estimate = 0.04, SE = 0.02, 95% CI [0.015, 0.064]), supporting Hypothesis 1.

**Table 3:** Results for path analyses in current study.

Independent variables	Dependent variables		
	Death anxiety *B* (SE) [95% CI]	Personal control *B* (SE) [95% CI]	Academic engagement *B* (SE) [95% CI]
Mortality salience	0.33^***^ (0.02) [0.286, 0.366]	−0.27^***^ (0.03) [−0.324, −0.216]	0.04 (0.03) [−0.013, 0.086]
Death anxiety			0.12^***^ (0.04) [0.046, 0.193]
Personal control			−0.08^**^ (0.03) [−0.129, −0.024]
Academic engagement (*p*)			0.03 (0.04) [−0.042, 0.095]
Gender	−0.33 (0.20) [−0.723, 0.057]	−0.28 (0.27) [−0.818, 0.247]	0.02 (0.10) [−0.169, 0.223]
Age	0.10^**^ (0.04) [0.018, 0.185]	−0.01 (0.06) [−0.129, 0.100]	0.01 (0.02) [−0.035, 0.044]
Residual variance at Level 1	0.65^***^ (0.03) [0.597, 0.717]	1.24^***^ (0.06) [1.127, 1.355]	0.80^***^ (0.04) [0.737, 0.880]
Residual variance at Level 2	0.63^***^ (0.11) [0.457, 0.887]	1.20 ^***^ (0.20) [0.878, 1.666]	0.01^*^ (0.01) [0.000, 0.016]

*N* = 102, *n* = 1,006. Mplus output provided the *p* values as one-tailed in Bayesian analyses. Academic engagement (*p*) = academic engagement on the previous day. ^*^p < 0.05, ^**^p < 0.01, ^***^p < 0.001 (one-tailed).

**Table 4:** The mediating role of death anxiety and personal control.

Effect Path	Estimate	SE	95% CI
Ind1: Mortality salience → Death anxiety → Academic engagement	0.04	0.01	[0.015, 0.064]
Ind2: Mortality salience → Personal control → Academic engagement	0.02	0.01	[0.006, 0.036]
Difference (Ind1–Ind2)	0.02	0.02	[−0.012, 0.050]
Direct effect	0.04	0.03	[−0.013, 0.086]
Total effect	0.10	0.02	[0.052, 0.139]

Daily mortality salience was negatively related to personal control (*B* = −0.27, SE = 0.03, 95% CI [−0.324, −0.216]). Personal control was negatively related to academic engagement (*B* = −0.08, SE = 0.03, 95% CI [−0.129, −0.024]). The mediating role of personal control on the relationship between mortality salience and academic engagement was significant (Estimate = 0.02, SE = 0.01, 95% CI [0.006, 0.036]), supporting Hypothesis 2. This pattern reflects a compensatory sequence: mortality salience was associated with diminished personal control (*B* = −0.27), which, in turn, was linked to increased academic engagement (*B* = −0.08). The negative sign for the latter path reflects the core compensatory mechanism—reduced control motivates greater academic engagement as a restorative strategy.

**Figure 2:**
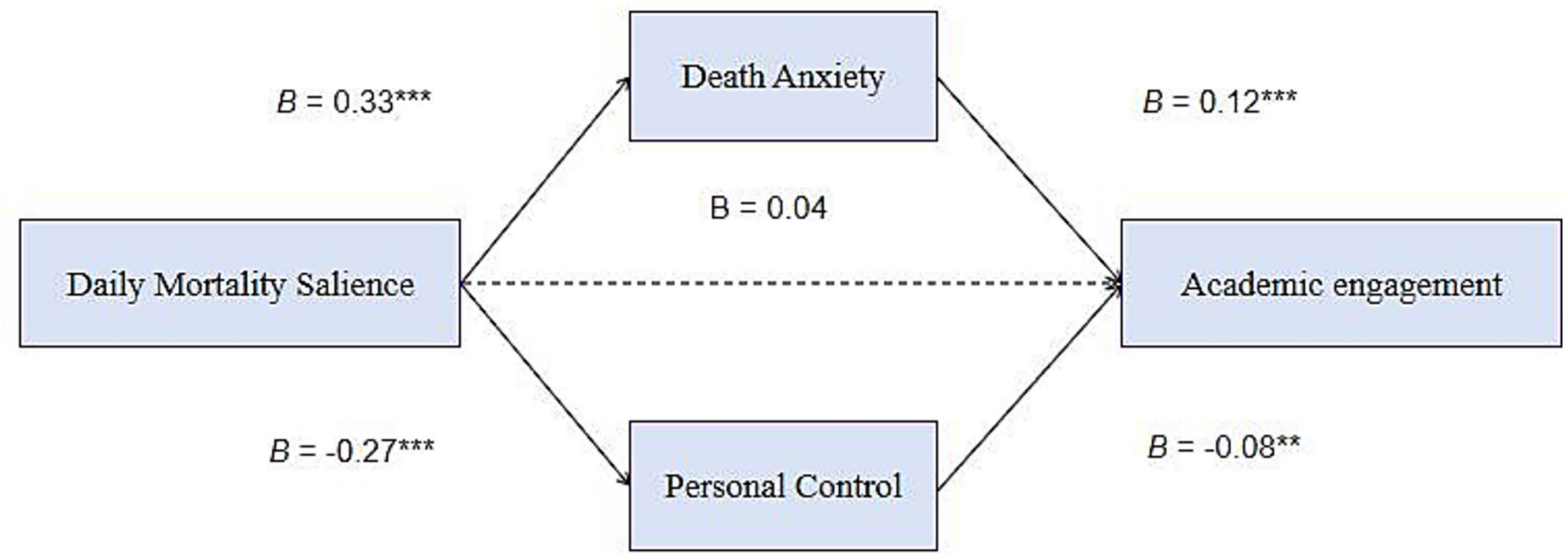
Results of the multilevel path analysis. ^*^p < 0.05, ^**^p < 0.01, ^***^p < 0.001 (one-tailed).

The path coefficients for the model are presented in Figure 2. In Table 4, we also provide the estimates of direct effect, total effect and difference of two indirect paths.

## 4 Discussion

Our daily diary study demonstrates that real-life mortality salience can enhance college students’ academic engagement through the parallel mediating pathways of death anxiety and personal control. This finding aligns with existing literature positing that immersion in meaningful activities is an effective strategy for mitigating negative states (Rosenzweig, 2016; Yu, 2020). Our research extends this understanding by specifying the motivational processes involved, showing that Chinese college students respond to mortality cues with increased learning-related enthusiasm and dedication, thereby confirming the role of academic engagement as an adaptive existential response.

It is important to consider the magnitude of these effects. The indirect pathways, while statistically significant, were modest in size. This is consistent with the nature of daily diary research, which captures within-person fluctuations and often yields effect sizes that are smaller than those found in between-person or experimental designs, yet are meaningful for understanding daily psychological processes (Nezlek, 2012). The argument follows that the cumulative effect of such daily nudges in engagement over an academic term could be substantively significant. However, our study focused on engagement as an outcome and did not assess other potential consequences of elevated daily death anxiety, such as increased general anxiety or distress. Therefore, while our model identifies a constructive compensatory pathway, the overall net impact of frequent mortality reminders on a student’s holistic wellbeing requires further inquiry.

This study also addresses a central theoretical debate concerning the primary motivator of mortality salience effects. While TMT identifies death anxiety as the core driver (Pyszczynski et al., 2004), other scholars contend that the loss of personal control is the fundamental mechanism (Van den Bos, 2009; Hogg et al., 2010). For example, Fritsche et al. (2008) found that individuals under normal mortality salience had a stronger need for personal control than those who imagined suicide (without depriving them of the personal control). Our findings reveal that daily mortality salience concurrently triggers both death anxiety and diminished personal control, suggesting that these are co-occurring psychological responses rather than mutually exclusive explanations. Although the mediating effect of death anxiety was numerically stronger than that of personal control—potentially explaining the continued dominance of TMT—the difference was not statistically significant, indicating that both pathways are substantively important.

Furthermore, our findings broaden the scope of both TMT and CCM. Prior research has documented positive outcomes of mortality salience, including strengthened group identity, prosocial behavior, and work engagement (Routledge et al., 2010; Castano, 2004; Dunn et al., 2020). We contribute to this line of inquiry by identifying academic engagement as another constructive consequence. For students, academic striving constitutes a primary avenue for self-validation, thereby serving a death-anxiety-buffering function. Simultaneously, we extend CCM by showing that academic engagement functions as an effective restorative strategy when personal control is threatened. Consistent with findings that control deprivation promotes self-regulation (Kay et al., 2008; McGregor et al., 2009), we demonstrate that academic engagement—through which individuals display competence and foster supportive relationships—successfully restores a sense of agency.

Beyond these immediate theoretical implications, it is crucial to situate the psychological processes examined here within a broader developmental ecology. University students concurrently navigate various adjustment challenges. Recent syntheses indicate that family dynamics, such as helicopter parenting, constitute a notable risk factor for increased anxiety, depression, and stress among college students, with effects often mediated through reduced self-efficacy and autonomy (La Rosa et al., 2025). This established context of psychosocial vulnerability likely modulates how students perceive and respond to daily existential threats. Therefore, the compensatory pathways identified in our study may vary in their accessibility or strength depending on such pre-existing developmental histories.

## 5 Implications

The findings of this study, which highlight how daily mortality reminders can motivate academic engagement through pathways of meaning-seeking (death anxiety) and agency-reinforcement (personal control), offer practical insights for educators. These insights resonate strongly with the dual emphases of contemporary Chinese educational initiatives: ‘Life Education’, which focuses on understanding death and cultivating reverence for life, and ‘Curriculum Ideology and Politics’, which aims to infuse learning with agency and value. Building on this alignment, educators can design targeted programs that not only encourage students to acknowledge the finitude of life and cherish learning opportunities but also explicitly frame academic engagement as a meaningful pathway to cultivating personal legacy, value, and a sense of control. By integrating these perspectives into pedagogical approaches—such as through project-based learning that addresses real-world challenges, reflective writing on personal growth and social responsibility, and autonomy-supportive classrooms that foster agency—educators can enhance students’ experiences of purpose and self-worth. Such strategies not only promote academic engagement but also equip students with psychological resources to constructively respond to existential concerns and adversity in future life, aligning with the broader educational goals of fostering moral, intellectual, and psychological development.

## 6 Limitations and future direction

This study has several limitations that warrant consideration. First, the generalizability of our findings is constrained by the cultural context, as our sample consisted exclusively of Chinese college students. Future research should test the cross-cultural validity of our model in diverse populations. Second, while we identified parallel mediating pathways through death anxiety and personal control, we did not examine the boundary conditions that determine when mortality salience preferentially triggers one pathway over the other. Future studies could employ experimental designs to investigate potential moderators (e.g., self-concept clarity or trait anxiety) that might influence these distinct psychological processes. Third, as participants voluntarily enrolled in a study on academic engagement, our sample may overrepresent students with preexisting positive attitudes toward learning. Subsequent research should explore how individual differences such as achievement motivation, academic resilience, or growth mindset moderate the observed relationships. Fourth, while we identified two compensatory pathways leading to academic engagement, we did not measure potential negative affective states (e.g., general anxiety, rumination) that might also be triggered by mortality salience. Future research should employ more comprehensive assessments to evaluate the potential trade-offs and net impact of mortality awareness on student wellbeing. Fifth, while our measure of daily mortality salience demonstrated good reliability (*α* = 0.91) in our study, its brevity is a limitation. It may not capture the full depth or intensity of death-related contemplation. Future research could incorporate multi-item scales or implicit measures on a subset of days to enrich the assessment.

In addition, the directionality of the observed daily associations warrants careful consideration. While our model and diary design (with predictors measured earlier in the day) are consistent with the proposed direction—from daily mortality salience to academic engagement—reverse or reciprocal causation cannot be ruled out. It is possible that students who are more deeply immersed in certain academic fields (e.g., medicine, literature, philosophy) or in a state of focused contemplation might encounter or become more attentive to mortality-related cues. Although our analyses controlled for the previous day’s level of engagement, our design does not permit strong causal inferences. Future research could employ experimental manipulations of mortality salience or cross-lagged panel designs across longer timeframes to better disentangle the temporal and potentially reciprocal relationships between these daily experiences.

## 7 Conclusion

Based on a 10-day daily diary study, this research demonstrates that daily mortality salience positively influences college students’ academic engagement through two parallel psychological mechanisms: heightened death anxiety and threatened personal control. These findings integrate TMT and CCM by revealing that academic engagement serves as a compensatory strategy to manage existential threat—both by alleviating death anxiety through the pursuit of symbolic immortality and by restoring a sense of control through self-regulation and achievement. The study not only advances theoretical understanding of how individuals cope with mortality awareness but also offers practical insights for designing educational interventions that foster resilience and meaning in learning.

## Data Availability

The raw data supporting the conclusions of this article will be made available by the authors, without undue reservation.
